# The Sodium Channel β4 Auxiliary Subunit Selectively Controls Long-Term Depression in Core Nucleus Accumbens Medium Spiny Neurons

**DOI:** 10.3389/fncel.2017.00017

**Published:** 2017-02-13

**Authors:** Xincai Ji, Sucharita Saha, Guangping Gao, Amy W. Lasek, Gregg E. Homanics, Melissa Guildford, Andrew R. Tapper, Gilles E. Martin

**Affiliations:** ^1^Department of Psychiatry, The Brudnick Neuropsychiatric Research Institute, University of Massachusetts Medical School, WorcesterMA, USA; ^2^Gene Therapy Center, University of Massachusetts Medical School, WorcesterMA, USA; ^3^Department of Psychiatry, University of Illinois at Chicago, ChicagoIL, USA; ^4^Department of Anesthesiology, University of Pittsburgh, PittsburghPA, USA

**Keywords:** nucleus accumbens, spike-timing-dependent plasticity, sodium channel, Scn4b, knockout mice, calcium imaging, dendrites, long-term depression

## Abstract

Voltage-gated sodium channels are essential for generating the initial rapid depolarization of neuronal membrane potential during action potentials (APs) that enable cell-to-cell communication, the propagation of signals throughout the brain, and the induction of synaptic plasticity. Although all brain neurons express one or several variants coding for the core pore-forming sodium channel α subunit, the expression of the β (β1–4) auxiliary subunits varies greatly. Of particular interest is the β4 subunit, encoded by the Scn4b gene, that is highly expressed in dorsal and ventral (i.e., nucleus accumbens – NAc) striata compared to other brain regions, and that endows sodium channels with unique gating properties. However, its role on neuronal activity, synaptic plasticity, and behaviors related to drugs of abuse remains poorly understood. Combining whole-cell patch-clamp recordings with two-photon calcium imaging in Scn4b knockout (KO) and knockdown mice, we found that Scn4b altered the properties of APs in core accumbens medium spiny neurons (MSNs). These alterations are associated with a reduction of the probability of MSNs to evoke spike-timing-dependent long-term depression (tLTD) and a reduced ability of backpropagating APs to evoke dendritic calcium transients. In contrast, long-term potentiation (tLTP) remained unaffected. Interestingly, we also showed that amphetamine-induced locomotor activity was significantly reduced in male Scn4b KO mice compared to wild-type controls. Taken together, these data indicate that the Scn4b subunit selectively controls tLTD by modulating dendritic calcium transients evoked by backpropagating APs.

## Introduction

It has long been established that adaptation of organisms to their environment depends in part on neuron-to-neuron propagation of action potentials (APs) throughout the brain. More recently, research has provided clear evidence that APs, upon retrograde propagation into the dendritic compartment, also control induction of synaptic plasticity by interacting in a precise time-dependent fashion with excitatory post-synaptic potentials (Caporale and Dan, 2008; Sjostrom et al., 2008). In a previous work, using spike-timing-dependent plasticity (STDP) as a stimulation paradigm to induce synaptic plasticity, we showed that tLTP was tightly regulated by NMDA receptors while backpropagating APs and calcium-induced calcium release were key factors controlling tLTD (Ji and Martin, 2012). APs require the participation of sodium channels that control the initial fast rise of the membrane potential following an initial depolarization. These channels are formed by the association of a large pore-forming α subunit with accessory proteins or β subunits (Catterall, 2000). Although a single α subunit forms the core of the channel and is functional on its own (Barchi, 1988), each of the various β subunits (β1–4) modulates α subunit gating properties in unique ways (Brackenbury and Isom, 2011). Of particular interest is the β4 subunit, encoded by the Scn4b gene, as it prevents normal inactivation, conferring channels the ability to evoke a resurgent current upon repolarization (Grieco et al., 2005; Aman et al., 2009; Bant and Raman, 2010). While all brain neurons express one or more variants of the gene coding for the sodium channel α subunit (Goldin et al., 2000; Goldin, 2001), those expressing Scn4b are restricted to discreet brain regions particularly the dorsal striatum and the NAc (Oyama et al., 2006; Miyazaki et al., 2014), a structure known for mediating drugs of abuse’s rewarding properties (Feltenstein and See, 2008). To date, the role of Scn4b beyond controlling sodium channel gating properties (Grieco et al., 2005; Aman et al., 2009; Huth et al., 2009; Bant and Raman, 2010) and spine density in hippocampal cultured neurons (Oyama et al., 2006) remains unclear. Here, we investigated its functional role in regulating synaptic plasticity in core NAc medium spiny neurons (MSNs). Using Scn4b shRNA and knockout mice (KO), we tested the hypothesis that manipulations of Scn4b expression in core NAc MSNs selectively impacts tLTD by modifying properties of APs, and by extension their ability to drive calcium signals in dendrites. We report that alterations of Scn4b expression significantly shifted APs threshold. This effect was associated with a large reduction of the probability of MSNs to induce tLTD, but not tLTP, in Scn4b KO and KD mice, irrespective of D1 dopamine receptor expression. Two-photon calcium imaging showed that backpropagating AP-driven calcium signals was attenuated in second order dendrites of KO mice compared to wild type animals. Also, given the well-established role of the accumbens in mediating amphetamine-induced locomotor activity (Kelly et al., 1975; Pijnenburg et al., 1976), we tested whether alteration of the balance between tLTP and tLTD in mice lacking the Scn4b subunit impacted the effects of amphetamine on this behavior. Taken together, these data suggest that the Scn4b subunit selectively controls tLTD formation by attenuating dendritic calcium transients following alteration of APs, effect that may be necessary for the psychostimulant action of amphetamine.

## Materials and Methods

B6.Cg-Tg(Drd1a-tdTomato) mice from Jackson laboratory, and Scn4b KO mice (described below), were housed in an animal care facility approved by the American Association for the Accreditation of Laboratory Animal Care (AAALAC). We backcrossed B6.Cg-Tg(Drd1a-tdTomato) mice into the C57Bl/6 background. Animal experimental procedures were approved by the Institutional Animal Care and Use Committee of University of Massachusetts Medical School and the University of Pittsburgh. All efforts were made to minimize animal suffering and to reduce the number of animals used. Mice were housed four per cage and maintained at constant temperature and humidity with a 12-h light–dark cycle. Water and food were provided *ad libitum*.

### Targeting Vector Construction

A 9745 bp Strain 129S7AB2.2 genomic DNA fragment harboring a portion of the Scn4b locus was subcloned from a BAC clone obtained from Source Bioscience (Nottingham, UK) into pStart-K using recombineering as described (Wu et al., 2008). Recombineering was also used to insert loxP/SalI sequences 199 bp upstream of Exon 2 and ScaI/loxP 266 bp downstream of Exon 2. SalI/frt flanked PGK-Neo derived from pK11 (Meyers et al., 1998) was subsequently subcloned into the SalI site that was introduced upstream of Exon 2. Gateway recombination was then used to move the modified Scn4b genomic DNA insert into pWS-TK3 (Wu et al., 2008). The targeting construct was linearized with NotI for gene targeting. This construct included 5′ and 3′ arms of homology that were 4.1 and 5.0 kb, respectively, and the floxed portion of the Scn4b locus was 647 bp.

### Gene Targeting in ES cells

The linearized targeting construct was electroporated into R1 (Nagy et al., 1990) mouse embryonic stem (ES) cells as previously described (Homanics et al., 1997). G418 and gancyclovir resistant ES cell clones were screened for gene targeting by Southern blot analysis with EcoRI and a 5′ external probe (Scn1+2) as illustrated in **Supplementary Figure S1**. Targeted clones were further analyzed with the following enzyme/probe combinations: EcoRV/Scn1+2; NdeI/Scn3+4; ScaI/Scn3+4; SpeI/Scn3+4. Note that the ScaI analysis was also diagnostic for the presence/absence of the 3′ loxP site. Scn1+2 is a 576 bp probe amplified (Primer 1 = 5′-ACTCCCCCGTGAACTCTTCT-3′; Primer 2 = 5′-TCTTACCCAAAAGCCCAGTG-3′) from mouse genomic DNA and binds to Exon 1. Scn3+4 is a 543 bp probe amplified (Primer 3 = 5′-TAAAAATTGCCCGTCTCCTG-3′; Primer 4 = 5′-GTGGAACATTCTCGCTGGTT-3′) from mouse genomic DNA and binds to Exon 5 between the 3′ end of the targeting construct and the NdeI site. The results presented here are derived from ES cell clone 254B5.

### Scn4b Knockout Mouse Production

Correctly targeted ES cell clones were microinjected into C57BL/6NCrl embryos (obtained as frozen embryos from Charles River) and subsequently implanted into pseduopregnant CD1 recipient females. Chimeric males were mated to C57BL/6J females. To create global KOs, F1 mice that were heterozygous for the floxed+neo locus were mated to EIIa-cre transgenic deleter mice (JAX stock #003724; C57BL/6 background). F2 heterozygous, cre recombined mice were mated to C57BL/6J mice to remove EIIa-cre from the pedigree. Mice were routinely genotyped by Southern blot analysis using NdeI and the Scn3+4 probe. Breeding pairs of cre recombined heterozygous (+/f) mice of the F3/N3 generation were shipped to UMass.

### Scn4b RT-PCR

**FIGURE 1:**
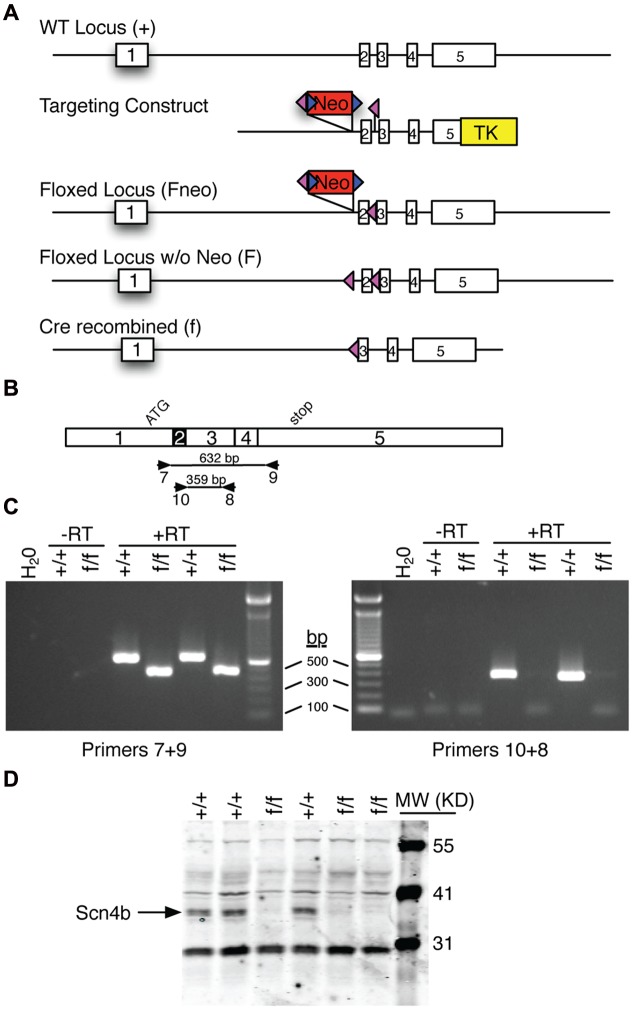
**Overview of Scn4b gene targeted mouse production.** **(A)** Diagram of the various Scn4b loci. The wild type (WT) locus was modified in ES cells using the DNA targeting construct to create the floxed locus that includes the neomycin resistance (Neo) gene. Following germline transmission of this targeted locus, FLPe-mediated recombination was used to selectively remove the Neo gene. Cre-mediated recombination was used to remove Exon 2 of the Scn4b gene. **(B)** Diagram of the Scn4b cDNA showing exon boundaries, the start of translation (ATG), the translation termination codon (stop), PCR primer binding sites (numbered arrows), and the predicted size of PCR amplicons. **(C)** Photographs of agarose gel analysis of RT-PCR products from homozygous WT (+/+) and global KO (f/f) mouse cortex. Also included are water and minus reverse transcriptase (-RT) controls. In the left panel, WT products are 632 bp as predicted. In contrast, products from f/f samples are 173 bp smaller as predicted following deletion of Exon 2. In the right panel, Primers 10+8 produced the 359 bp product only from WT samples; f/f samples did not amplify with this primer set. **(D)** Western blot of cerebellar extracts with a Scn4b antibody. Note that the ∼37 KD Scn4b protein is readily detected in +/+ samples. In contrast, no Scn4b is detected in f/f samples.

Wild type and global KO mice were sacrificed and tissue samples from cortex were isolated, flash frozen and stored at -80C until processed. Total RNA was isolated from samples using Trizol (Life Technologies, Inc.). Approximately 1 μg of RNA was converted to cDNA using an iScript cDNA Synthesis Kit (Bio-Rad Laboratories). Scn4b cDNAs were amplified using primers illustrated in **Figure 1B** (Primer 7 = AACCGAGGCAATACTCAGGCGAGA; Primer 8 = AACCACTTGGAGGAAGATGGTGGCA; Primer 9 = GGCAACCCGTTCTCTGTGTTGTCA; Primer 10 = TATCTGTGGGAAAGGCCACCACCA) and Platinum Taq DNA Polymerase High Fidelity (Life Technologies). PCR cycling parameters were 95C 2 min; 40 cycles of 95C 30 s, 60C 30 s, and 72C 90 s; and a final 72C 5 min extension. PCR products were analyzed on 2% agarose in TAE buffer with ethidium bromide.

### Scn4b Western Blot Analysis

Brains from 10 weeks-old WT (*n* = 3) to KO (*n* = 3) mice were dissected, the cerebellum removed, divided in half on the sagittal plane, and flash frozen. Membrane proteins were isolated from one half of each cerebellum using the Mem-PER Plus Extraction Kit (ThermoScientific, 89842). Protein lysates were concentrated through acetone precipitation and the concentration determined using a BCA assay. Thirty micrograms of protein lysate were loaded on a Novex 12% Tris-Glycine gel (Invitrogen), electrophoresed, and transferred onto nitrocellulose using an iBlot Transfer Device (Invitrogen). Membranes were blocked in 5%BSA/TBS for 1 h at RT and incubated overnight at 4°C with a primary monoclonal antibody (anti-SCN4b; UC Davis/NeuroMab facility, clone N168/6, #75-198) at a dilution of 1:4000 in 5%BSA/TBS+0.2% Tween 20. Membranes were subsequently incubated with an IgG1 secondary fluorescent antibody according to manufacturer’s instruction (Licor, #926-68050) and visualized using the Odyssey Infrared Imaging System (Licor Biosciences).

### Scn4b shRNA

The Scn4b short hairpin RNA (shRNA) construct was designed using the siDesign Center (GE Dharmacon). The 19 nucleotide siRNA targeting sequence was incorporated into a hairpin sequence and cloned into pLL3.7 as previously described (16). The sequence of the 19 nucleotide targeting sequence is: 5′-GACCCTAAGGTGAGAGT-GA-3′. The shRNA was able to knockdown (KD) *Scn4b* mRNA in cell culture by 78% (data not shown). For adeno-associated virus (AAV) production, the Scn4b shRNA was excised from pLL3.7 and cloned into an AAV9 transfer vector, JHUMCS, derived from the original L307 vector (17) but lacking the IRES-GFP sequence. The AAV shRNA plasmid, ScAAV9.CB.eGFP.U6-ShScn4b-268, was packaged into AAV by the University of Massachusetts Medical School vector core facility. Briefly, HEK293 cells were transfected with the transfer vector and three helper plasmids, pRSV-REV, pIVS-vesicular stomatitis G protein (VSVg), and pMDL gag/pol. Transfected HEK293 cells were grown in neurobasal media and 2 days later the media was collected, spun at 2000 r.p.m. for 5 min, and passed through a 0.45-mm filter. The vector was injected in the nucleus accumbens of 21 day-old B6.Cg-Tg(Drd1a-tdTomato) mice, and patch-clamp recordings were performed 3 weeks later.

### shRNA Knockdown Experiments

Drd1a-tdTomato mice were injected in the dorso-medial nucleus accumbens with virus and analyzed 3 weeks following injection. A number of MSNs in the vicinity of the injection site near the anterior commissure expressed GFP (**Supplementary Figure S2A**). At higher magnification, some GFP+ MSNs infected by the AAV9 virus are clearly visible (**Supplementary Figure S2A**, 40×). Among these GFP+ MSNs, some were also RFP+, indicative of dopamine D1 receptor expression (**Supplementary Figure S2A**, D1R+). qPCR performed on 1 mm wide disks of tissue containing the anterior commissure, the core accumbens landmark, showed that mRNA coding for the Scn4b subunit was significantly [*P* < 0.05, and *F*(2,2) = 36.05] down regulated by 25% in mice injected with the Scn4b shRNA-expressing virus compared to mice injected with the control virus (Control, **Supplementary Figure S2B**). This value may not entirely reflect the full extent of Scn4b mRNA down regulation, since our tissue samples contained a sizable fraction of MSNs that were not infected by the virus as shown at low magnification (Data not shown).

### Animal Surgeries and Slice Preparation

ScAAV9.CB.eGFP.U6-ShScn4b-268 construct was provided by Dr. E. Lasek and was packaged by UMass Medical School vector core facility. We anesthetized mice with a mixture of ketamine (100 mg/kg) and xylazine (10 mg/kg; VEDCO) before shaving and disinfecting the surgical area. Mice were placed in a stereotaxic frame (Stoelting Co., Wood Dale, IL, USA) and a small incision was cut in the scalp to expose the skull. Using Bregma and Lambda as landmarks, the skull was leveled in the coronal and sagittal planes. ScAAV9 virus (1 × 10^12^ viral particles/ml, 0.6 μl/side) was injected bilaterally into NAc core region (**Figure 1A**); anterior–posterior (AP), +1.5 mm from bregma; medial–lateral (ML), ± 1.2 mm from midline; dorsal–ventral (DV), -6.5 mm from brain surface. The virus was injected at a flow rate of 0.1 μl/min. The injection needle remained in place for 5 min post-injection before slowly being withdrawn. Mice were then returned to their home cages for 21 days before electrophysiology experiments.

### Slice Preparation and Electrophysiology

To prepare slices from fresh brain tissue, we rapidly removed and transferred the brain in a cold (∼ +0.5°C) oxygenated (95% O_2_ and 5% CO_2_) cutting solution of the following composition (in mM): 95 *N*-methyl-D-glucamine (NMDG), 2 thiourea, 5 Na^+^-ascorbate, 3 Na^+^-pyruvate to cut 200 μm-thick slices transversely with a Vibroslicer (VT1200, Leica MicroInstrutments; Germany). Slices were immediately transferred in an incubation chamber and left to recuperate in the NMDG-based solution for 22 min at 32°C before being moved into a chamber containing an artificial cerebrospinal fluid (ACSF; in mM): 126 NaCl, 2.5 KCl, NaH_2_PO_4_.H_2_O, 1 MgCl_2_, 2 CaCl_2_, 26 NaHCO_3_, 10 D-Glucose, at room temperature. Slices were left in this chamber for at least 1 h before being placed in a recording chamber and perfused with ACSF at a constant rate of 2–3 ml/min at room temperature (∼21°C). We visualized neurons in infrared differential interference contrast (60×, IR-DIC) video microscopy using a fully motorized microscope (Scientifica; England). We performed whole-cell patch clamp recordings. Briefly, we filled borosilicate glass electrodes (1.5 mm OD, 4–6 MΩ resistance) with an internal solution containing (in mM): 120 K-methanesulfonate, 20 KCl, 10 Hepes, 2 K_2_ATP, 2 K_2_GTP, and 12 phosphocreatine. Following seal rupture, series resistance (Rs 10–20 MΩ) was fully compensated in current-clamp recording mode, and periodically monitored throughout recording sessions. Recordings with Rs changes larger than 20% were rejected. We acquired voltage and current traces in whole-cell patch-clamp with an EPC10 amplifier (HEKA Elektronik; Germany). We sampled and filtered voltage and current traces acquired with PatchMaster 2.15 (HEKA Elektronik) at 10 and 2 kHz, respectively. We subsequently analyzed all traces off-line using FitMaster 2.15 (HEKA Electronik). To generate synaptic plasticity, we paired post-synaptic AP (evoked with a 5 ms/6–800 pA depolarizing pulse) followed by EPSP with a 20 ms interval at a rate of 1 Hz for 90 s as previously described (Ji and Martin, 2012). As previously described (Ji and Martin, 2012), we measured magnitude of synaptic plasticity by comparing EPSPs maximum amplitude in a 20 ms time window 10 ms before and after the onset of the stimulus. We performed this measurement on 30 consecutive EPSPs before, and 20 min after AP-EPSP pairing. We expressed the difference of EPSP amplitude before and after induction as percent of control (100%). We performed statistical analysis on STDP and AP threshold, in Prism 5 (Graphpad, La Jolla, CA, USA) running on a Mac Power PC G5, with a one-way ANOVA followed by a Dunnett’s *post hoc* test, with *P* < 0.05 considered statistically significant. All averaged results are expressed as mean ± SEM values.

### Two-Photon Laser Scanning (2PLSM) Calcium Imaging

We filled recording pipettes with Fluo5F (20 μM) and Alexa 549 (30 μM; Molecular Probes, Eugene, OR, USA). Dendritic locations were determined based on the loading of Alexa 594 after breaking into the cell (15–25 min). A custom-made 2PLSM (Scientifica, UK) with a Mai Tai HP Ti:Sapphire (Spectra-Physics, Santa Clara, CA, USA) laser providing pulses at <100 fs at 820 nm was used for imaging. Linescans along each dendritic location were performed using a 60X (1.0 NA) objective (Olympus). We recorded activity at various dendritic locations from the soma with clear branch points (1°= primary, 2° = secondary, 3° = tertiary). Calcium transients were acquired using a standardized protocol controlled by Scanimage (Vidrio) that allowed the laser beam to be “parked” between each image capture. We used PatchMaster 2.15 (Germany) to evoke theta burst firing patterns in response to 800 pA current injections to drive Ca^2+^ transients in MSNs. Only cells with stable resting membrane potential (RMP) between -82 and -90 mV were selected for recordings. All experiments were conducted at 32–34°C > 15 min after break in. The protocol consisted of three consecutive potentials at a frequency of 5 Hz, 10 s intervals, at 42 frames per second. Fluorescence ratios were calculated based on Δ*F* = *F*/*F*_0_, where the baseline fluorescence (*F*_0_) was the average of first 20 frames. Three responses were averaged and then smoothed using Savitzky–Golay (first–order polynomial, 15 pt) filtering to acquire the individual calcium traces for each dendritic location. Slope of the rise of the signal was calculated as shown in **Figure 4D**. Maximum amplitude of the signal was captured for each calcium trace, within a limited response window of 15 frames following the baseline fluorescence. Statistical analysis of differences in dendritic calcium transients were established using the non-parametric Mann–Whitney test, and were considered significant if *P* < 0.05. Error bars shown are SEM. All analysis was conducted using custom-written code in MATLAB (MathWorks).

### Behavior

Scn4b KO males (*n* = 8) and their wild-type litter mates (*n* = 6) were i.p. injected with 100 μL saline in their homecage for 2 days prior to the start of the experiment to habituate to injections. On the 3rd day, mice were placed in a novel cage situated in a locomotor activity apparatus (Photobeam Activated System, San Diego Instruments, San Diego, CA, USA) for 1 h to habituate to the cage and to measure baseline activity. Following the habitation period, mice were injected with saline and placed back in the cage for an additional hour of locomotor activity measurements (data not shown). On days 4 and 5, mice were again placed in a novel cage for an hour of habituation and baseline activity measurements before being injected with either saline or 5 mg/kg amphetamine (D-amphetamine sulfate in saline, Sigma-Aldrich, MI). Injections were cross-balanced across genotype and day. For each day, activity was measured as the number of beam breaks over the indicated time-course, with baseline defined as the total activity 10 min after the start of the experiment until injections, and the experimental measurements from 10 min post-injection until the end of the experiment. Experimental activity results are presented as a percent of baseline activity. We used a 2-way ANOVA and a *post hoc* analysis to test statistical significance.

## Results

To study the role of Scn4b on MSNs physiology, we used gene targeting (**Figure 1A**) and ES cell technologies to create mice in which Exon 2 of the Scn4b gene was flanked by loxP sites (i.e., floxed). Floxed mice were crossed with a cre general deleter mouse line to recombine the targeted locus and delete Exon 2. To verify that cre recombination disrupts the Scn4b gene, mRNA from cortex was analyzed by RT-PCR (**Figures 1B,C**) using two primer sets. Primers 7+9 which bind to Exons 1 and 5, respectively, revealed the expected 632 bp fragment from WT mice. In contrast, these primers produced a 459 bp from KO samples as predicted for deletion of the 173 bp Exon 2. Primers 8+10 which bind to Exons 3 and 2, respectively, revealed the expected 359 bp fragment only from WT samples. KO samples did not yield a PCR product with this primer set. This result is also consistent with deletion of Exon 2. Deletion of Exon 2 is predicted to create a frameshift mutation and result in a truncated Scn4b product that is 20 amino acids plus 9 non-sense amino acids. Full length Scn4b is 228 amino acids. Western blot analysis of cerebellar extracts using a Scn4b selective antibody confirmed the absence of the ∼37 KD Scn4b protein in KO samples (**Figure 1D**; **Supplementary Figure S1**).

### The Scn4b Subunit Expression Selectively Regulates the Expression of tLTD

**FIGURE 2:**
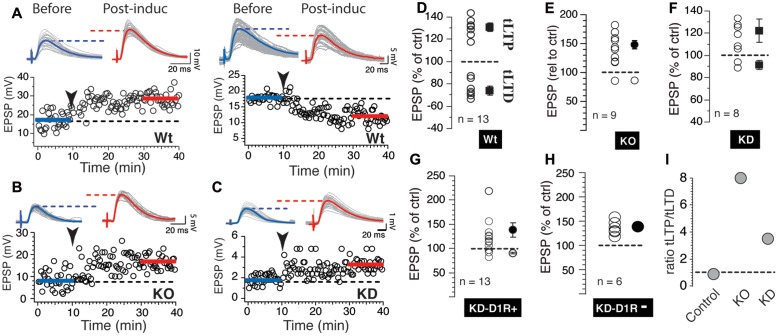
**Scn4b regulates induction of tLTD in NAc medium spiny neurons (MSNs).** **(A)** Light gray overlapping traces show 30 consecutive EPSPs before (before) and after (post-induct) AP-EPSP pairing in two representative individual NAc MSNs from Control mice showing tLTP (left panel) and tLTD (right panel). Blue and red traces show averaged EPSP amplitudes before and after induction. Horizontal dashed lines are visual aids to compare the average EPSP amplitudes. Below, from the same neuron, graph of EPSPs amplitude monitored every 20 s before and after synaptic plasticity, respectively. Arrowhead indicates time of induction. **(B,C)** Representative examples of tLTP recorded in KO (left panel) and KD mice (right panel). **(D)** Each symbol indicates change of EPSP amplitude measured 20 min after induction in individual MSN. Symbols above and below dashed line show tLTP and tLTD, respectively. Change of synaptic strength after induction is expressed as percent of control (100%). Solid symbols with SEM show averaged tLTP and tLTD. **(E,F)** As for panel **D** each symbol indicates change of synaptic strength in neurons recorded in KO and KD mice, respectively. **(G,H)** Plasticity in KD mice expressing D1R **(G)** or not **(H)**. **(I)** Ratio of the number of MSNs evoking tLTP/tLTD in Control, KO and KD mice.

In a previous study, we showed that accumbens tLTD is governed by somatic APs, a phenomenon that is blocked by intracellular calcium chelators, and L-type calcium channels antagonists (Ji and Martin, 2012). Given the key role of sodium channels in shaping APs, we hypothesized that Scn4b regulates the expression of tLTD by altering APs properties. In Control mice, 90 consecutive APs – EPSPs pairings (20 ms delay at 1 Hz) induced both tLTP (**Figure 2A**, left panel) and tLTD (**Figure 2A**, right panel) in equal proportion (tLTP, *n* = 6; tLTD, *n* = 7). While tLTP amplitude was 131.5 ± 3.67, that of tLTD was 69.33 ± 4.8% of control (**Figure 2D**), similar to what we previously reported in similar experimental conditions (Ji and Martin, 2012). In KO mice, the vast majority expressed a robust tLTP (151.8 ± 7.9%, *n* = 8; **Figures 2B,E**), while only one MSNs showed a weak depression (**Figure 2E**). In mice injected with AAV-shRNA (KD) targeting Scn4b expression (**Supplementary Figure S2**), tLTP was also the predominant form of plasticity (**Figures 2C,F**), while weak tLTD was observed in only two MSNs (93% of control, **Figure 2F**). In order to determine whether this effect was related to the expression of dopamine receptors, we also injected Scn4b shRNA in B6 DAR1-tdTomato BAC transgenic mice, and recorded from MSNs expressing (D1R+) or not (D1R-) dopamine D1 receptors. We found that the expression of D1 receptors has no clear influence on synaptic plasticity in shRNA mice as it was remarkably similar in both D1R+ and D1R- neuronal populations (**Figures 2G,H**). Thus, most MSNs D1R+ (*n* = 9) and D1Rs- (*n* = 6) MSNs evoked tLTP (137.71 ± 14.37; 136.56 ± 13.94% of control, respectively), tLTD (89.80 ± 2.33%) was observed in only four MSNs in D1R+ MSNs (**Figure 2H**), and none in D1R- MSNs (**Figure 2H**; *n* = 6). Statistical analysis shows a significance [*F*(2,45) = 5.15; *p* = 0.0096]. *Post hoc* analysis showed a significant difference of threshold in KO compared to wild type (*p* = 0.0054), but not between Wt and KD (*p* = 0.058). To quantify the probability of MSNs to induce tLTP and tLTD, we calculated the ratio of the number of tLTP/tLTD MSNs, with the number 1 indicating perfect parity between both forms of plasticity. In Control mice, the ratio was 0.85, pointing to a slightly higher probability of evoking tLTD over tLTP (**Figure 2I**). However, in Scn4b KO and KD mice, a ratio of 8 and 4, respectively, indicated a dramatic shift in favor of tLTP (**Figure 2I**).

**FIGURE 3:**
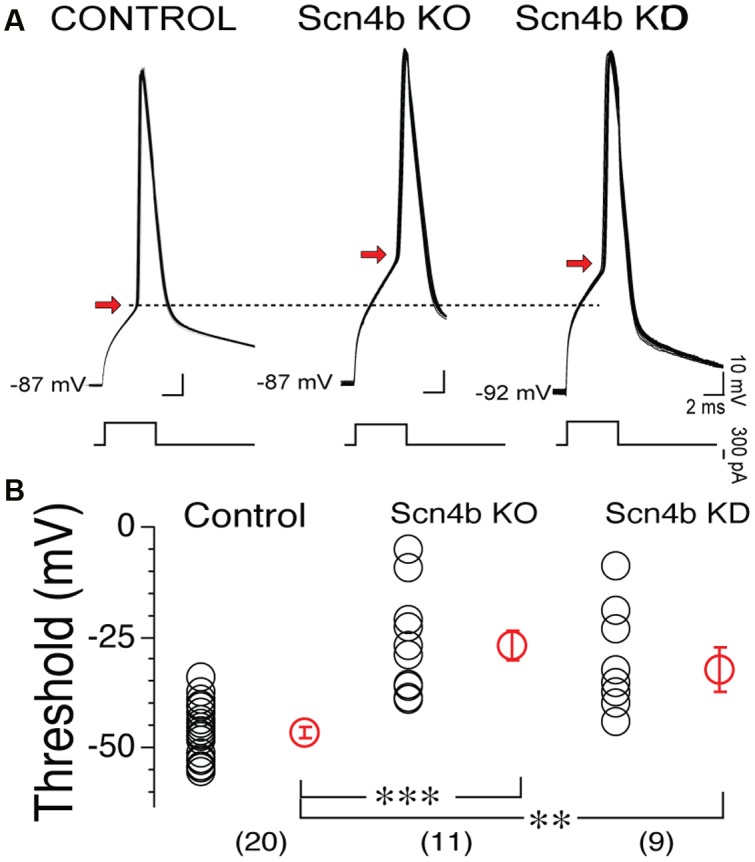
**The Scn4b subunit controls action potential (AP) threshold.** **(A)** Overlay of 90 consecutive representative APs recorded during induction of synaptic plasticity in Control, Scn4b KO and KD mice. AP threshold in each condition is indicated by red arrow. **(B)** Threshold values in control, in KO and KD mice. Each symbol represents an individual neuron. Red symbols with SEM show averages of threshold values. Numbers in parentheses below symbols indicate number of MSNs recorded in each condition.

Having shown that Scn4b expression controls the probability of tLTD induction, we set out to determine whether this effect was associated with changes in AP properties, the main factor contributing to tLTD formation (Ji and Martin, 2012). **Figure 3A** shows side-by-side representative examples of 90 overlaid APs recorded during induction in control, Scn4b KO and KD mice. In Scn4b KO and KD animals, AP threshold (red arrowhead) was noticeably more depolarized compared Control mice (**Figure 3A**). Averaging threshold values in these various experimental conditions confirmed this initial observation. Thus, AP threshold in Control mice was -46.39 ± 3.69 mV (**Figure 3B**, control; *n* = 20). In Scn4b KO mice (*n* = 11) and KD mice (*n* = 9), it was markedly shifted toward less negative potential of -26.69 ± 3.27 mV and -32.11 ± 4.91 mV, respectively (**Figure 3B**). A one-way ANOVA statistical analysis showed a significant difference of threshold between Wt, KO, and KD [*F*(2,42) = 20.9; *p* < 0.0001]. *Post hoc* analysis showed a significant difference of threshold in KO and KD mice compared to wild type with *p*-values of 0.0001 and 0.002, respectively (**Figure 3B**). Neither MSNs RMPs nor their input resistance (Rin) were significantly affected in KO mice, while their current-voltage relationship was smaller in KO compared to wt mice (**Supplementary Figure S3**).

### Scn4b Expression Controls Backpropagating AP-Evoked Dendritic Calcium Signal

**FIGURE 4:**
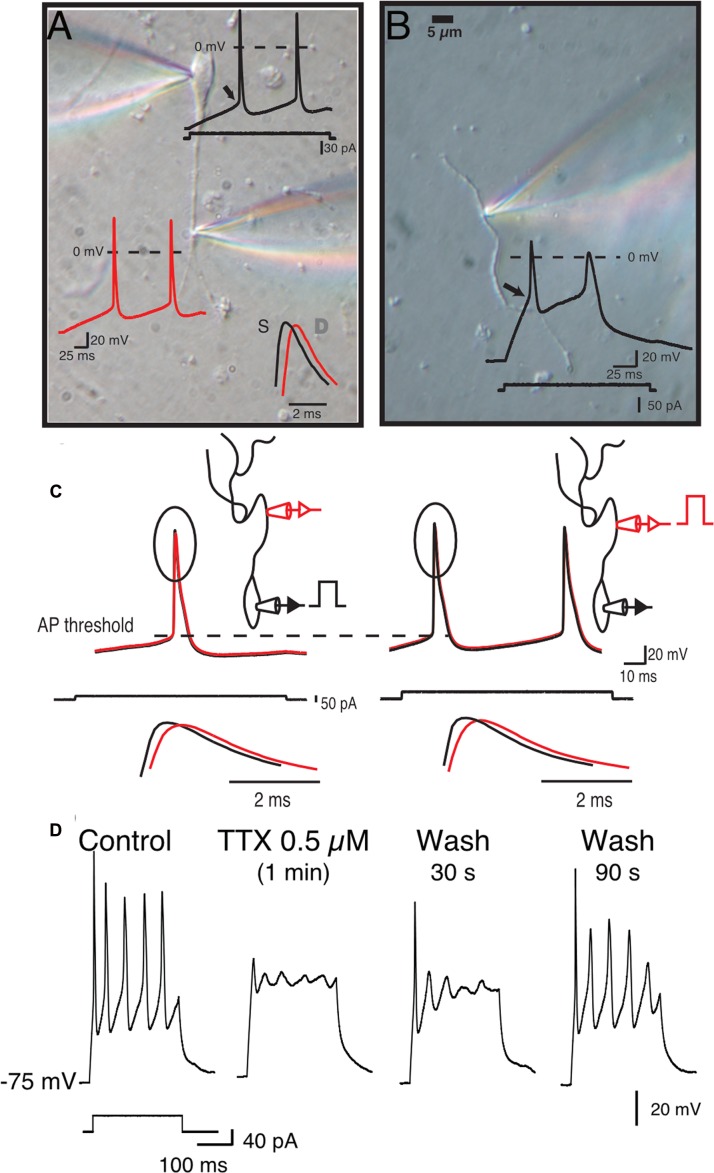
**Medium spiny neurons dendrites express sodium channels and backpropagating APs.** **(A)** Simultaneous double somatic and dendritic recordings on a freshly isolated MSN. Current step was injected in the soma and voltage was recorded in soma (black trace) and dendrite (white trace). Note the delay between the peaks of the first somatic and dendritic APs. **(B)** Voltage in response of a current step injected in an isolated dendrite. Note the much higher threshold (arrow head) and small overshoot (star) of dendritic APs. **(C)** Initiation of APs is somatic regardless of where the current step is injected (i.e., somatic left panels, or dendritic right panels). **(D)** APs recorded in isolated dendrites are totally and reversibly blocked by 0.5 μM TTX.

**FIGURE 5:**
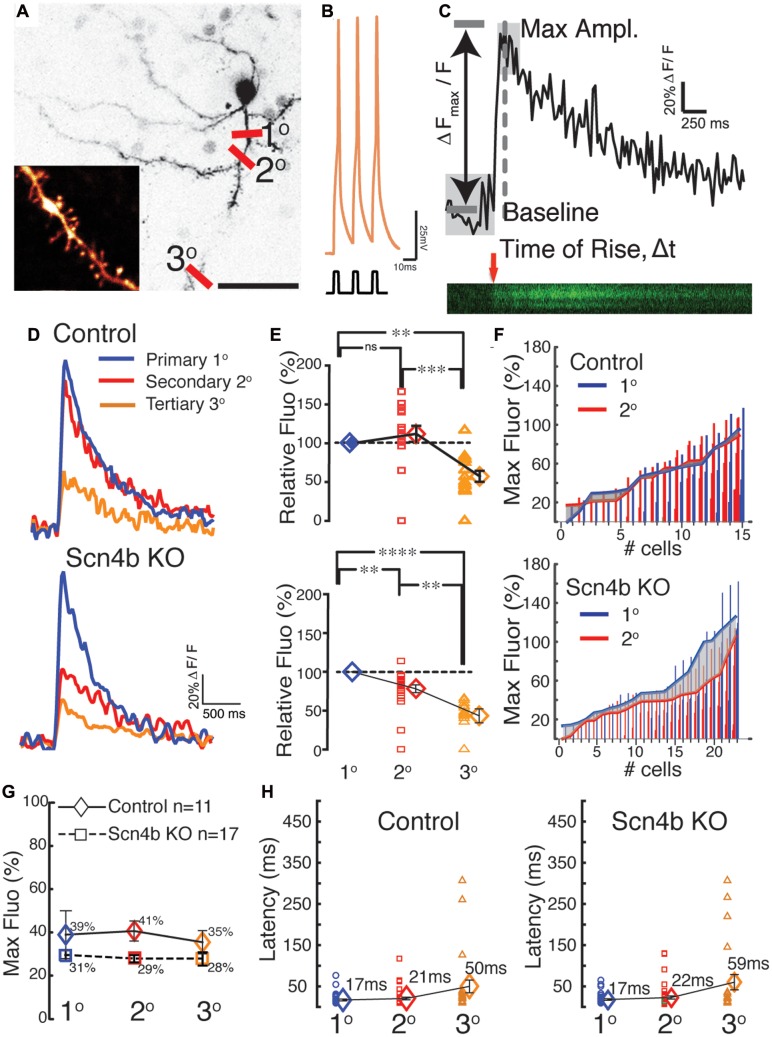
**Scn4b controls the amplitude of calcium signals evoked by backpropagating APs.** **(A)** Maximum intensity projection of soma and dendritic areas in a MSN. Red lines indicate the approx. dendritic sites (1°= primary, 2°= secondary, 3°= tertiary) where Ca^2+^ transients were measured as PLSM linescans for each MSN, which were visualized using Alexa 594. Scale = 30 μm. **(B)** Representative theta burst firing pattern in response to 800 pA current injections used to drive the Ca^2+^ transients in MSNs (RMP = -85mV). **(C)** A representative raw Ca^2+^ trace from three linescans from a 2° dendritic site expressed as ΔF_max_/F. Calculations for rising slope and maximum amplitude were based on the *t*-value and the average of the baseline signal. **(D)** Representative S-G filtered Ca^2+^ response recorded in 1°, 2°, and 3° dendrites resulting from a theta burst in Control (top panel) and KO mice (bottom panel). **(E)** Amplitude of Ca^2+^ transients in 2° (red symbols), and 3° dendrites (orange symbols) normalized to calcium responses measured in 1° dendrites (100%; blue symbols) in Control (top graph) and KO mice (bottom graph). Each symbol represents a neuron. **(F)** Maximum amplitude of Ca^2+^ responses from 1° (blue) to 2° (red) dendritic shown in ascending order in individual cells. The relationship of the Ca^2+^ signal between primary and secondary dendrites is shown by the solid lines which represents the average max amplitude of Ca^2+^ responses of the dendritic locations. The shaded area highlights the incongruity of the Ca^2+^ signal, which is more pronounced in the larger Ca^2+^ transients of the Scn4b KO in comparison to control animals where the transients are closely matched between 1° and 2° dendrites. **(G)** Mean values of maximal Ca^2+^ transients measured in spines expressed as percent of calcium transients in the corresponding dendritic shaft in Control (diamonds) and KO mice (Squares). Red triangles show averages. **(H)** Latency of calcium maximal fluorescence responses in 1°, 2°, and 3° dendrites. Latencies indicate that measurements were collected at similar distances along dendrites in both the Control and Scn4b KO groups.

Although our data suggest that the Scn4b subunit controls tLTD induction, how the former influences the latter was unclear. One putative path connecting Scn4b/APs and tLTD was calcium. In freshly isolated MSNs, as somatic APs invade dendrites (**Figures 4A,C**), through an active process due in part to the expression of TTX-sensitive sodium channels in MSNs dendrites (**Figures 4B,D**), they generate calcium transients (Plotkin et al., 2013). Having shown that AP-driven calcium-induced calcium release during synaptic induction is necessary for tLTD (Ji and Martin, 2012), we tested whether Scn4b expression controls the ability of APs to drive dendritic calcium transients. Combining two-photon laser scanning microscopy with patch clamp recordings, we measured calcium signals, driven by theta burst APs evoked somatically (**Figure 5B**), along the dendritic arbor in first (1°), second (2°), and third (3°) order dendrites (**Figure 5A**). Dendritic calcium signals, a proxy for measuring the strength of backpropagating APs, were characterized by a rapid rise followed by a slow decay (**Figure 5C**). The decay time course of the transients, where the ΔF returned to basal levels, (**Figure 5D**) was relatively indistinguishable when comparing dendritic arborizations (Control 1°: 2.97s, 2°: 2.96s, 3°: 2.62s, KO 1°: 3s, 2°: 2.65s, 3°: 2.37s), with small changes in KO mice that were not statistically significant. In Control mice, amplitude of calcium transients remained relatively stable, increasing slightly to 112.1 ± 10.37% in 2° dendrites relative to 1° processes (**Figure 5E**, top graph, red symbols). However, we observed a significant decline in 3° dendrites as the calcium signal dropped to 57.4 27 ± 7.08% (**Figure 5E**, orange symbols). Statistical analysis revealed a significant difference between 2° and 3° (*p* = 0.00082) and 1° and 3° dendrites (*p* = 0.0024) only. In KO mice, although the calcium signal similarly decreased between the 1° and 3° dendrites, the drop of amplitude was already detectable in 2° dendrites compared to 1° dendrites (78.22 ± 5.04%, **Figures 5E,F**, lower panels), an effect that was significant (*p* = 0.0108), unlike in Control mice (*p* = 0.092). The amplitude of calcium transients further declined in 3° processes (43.41 ± 8.97%; **Figure 5E**, orange symbols, *p* = 0.0086). The distribution of responses across cells shows that the attenuation of calcium transients (**Figure 5F**, gray area) in second order dendrites (**Figure 5F**, red line) of KO mice was mostly observed in 1° dendrite where calcium transients were stronger (gray area). Although calcium transients were robustly attenuated between dendritic shaft and spine heads (no attempts were made to distinguish between the different spine morphologies) in 1°, 2°, and 3° dendrites in both control and KO mice, a statistical analysis showed no difference between the two conditions (**Figure 5G**). **Figure 5H**, showing the latency between APs and calcium signals onsets in control and KO mice, indicates that changes of the amplitude of calcium signals are unlikely the result of transients being recorded at different distances from the soma. Finally, in shRNA mice, the calcium signal was also significantly depressed compared to Control in all dendritic compartments (not shown).

### Amphetamine-Induced Locomotor Activity Is Reduced in Scn4b KO Mice

**FIGURE 6:**
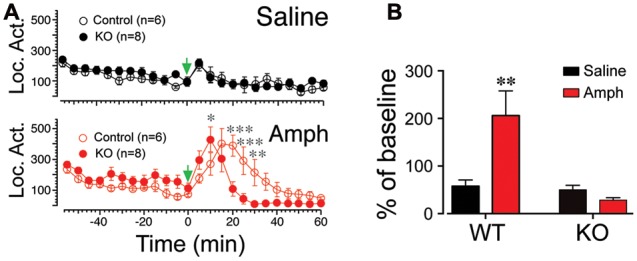
**The Scn4b subunit blocks amphetamine induced locomotor activity.** **(A)** Locomotor activity monitored every 5 min 1 h before and after amphetamine injection in wt (control, black symbols) and KO mice (red symbols). Arrowhead indicates time of injections. **(B)** Average locomotor activity in wt and KO mice following a single injection of saline (black columns) or amphetamine (gray columns). Note the robust inhibition of the activity in KO mice following amphetamine injection.

The accumbens is a key region mediating psychostimulants-evoked locomotor activity (Kelly et al., 1975; Pijnenburg et al., 1976). Considering the robust expression of Scn4b in this brain region, we tested whether its absence and the lack of tLTD affected KO mice response to amphetamine. In wild type (Control) and KO mice, locomotor activity remained stable, rising only slightly after injection of saline in both groups (**Figure 6A**, top graph). However, it rose sharply and reached a maximum 15–20 min following a single injection of 5 mg/kg amphetamine (green arrowhead, **Figure 6A** lower panel), before returning to control levels 50 min post-injection (**Figure 6A**, open red symbols). In KO mice (*n* = 8), while the locomotor activity also rose during the first few minutes following the injection, it peaked (i.e., 10 min post-injection) and declined much faster within 20 min (**Figure 6A**, KO, solid red circles). A two-way ANOVA revealed a significant main effect of time [*F*(23,276) = 15.39; *p* < 0.0001] and a significant time × genotype interaction [*F*(23,276) = 6.629; *p* < 0.0001]. *Post hoc* analysis showed a significant difference of amphetamine on locomotor activity in KO mice compared to wild type at 10, 20, 25, and 30 min (**Figure 6B**). Also, averaged over 1 h post-amphetamine injection, locomotor activity was significantly reduced in KO compared to wild type mice (*p* = 0.003; Amph, **Figure 6B**). Activity of wild type and KO mice were similar following saline injection (**Figure 6B**, Saline).

## Discussion

We provide for the first time, evidence indicating that low or no Scn4b expression dramatically impairs the ability of NAc MSNs to evoke tLTD, a consequence of attenuated dendritic calcium transients. More specifically, in absence of the subunit, APs evoked during induction of plasticity are characterized by a large shift of the AP threshold. Functionally, the ability of APs to drive calcium transients in dendrites is attenuated in KO mice, an effect mostly observed in secondary dendrites. Finally, the locomotor stimulating properties of amphetamine was significantly attenuated in KO mice.

Although there is still little known about the contribution of Scn4b on the sodium channel biophysical properties, some evidence suggest that the channel 1/2 max voltage activation is in part under the control of this subunit. Thus, in HEK cells, the voltage dependence of activation of peak sodium channel core subunit Na_v_1.1 was markedly shifted to the right (i.e., toward less negative potentials) in absence of the subunit, while the channel inactivation remains unaffected (Aman et al., 2009), confirming a previous study in tsA-201 cells transfected with the Na_v_1.2 core α subunit (Yu et al., 2003). These findings could explain in part the shift of AP threshold reported in our study. However, because this shift in HEK cells is noticeably smaller (i.e., +3–6 mV) than the change of threshold in MSNs from KO mice (i.e., +20 mV), it is unlikely that the lack of Scn4b alone accounts for such difference. A number of additional factors may influence the threshold value, among them changes in the ion conductance as well as density of voltage-gated sodium and potassium that can either raise or lower value of threshold (Trautwein, 1963). MSNs may compensate the loss of Scn4b by overexpressing the Scn2b, an auxiliary subunit commonly expressed in the striatum unlike Scn1b and 3b, possibly conferring biophysical properties to sodium channels unseen in wild type animals. However, *in situ* hybridization experiment showed that Scn2b expression only marginally increased in Scn4b KO mice (Miyazaki et al., 2014). Another possibility may involve potassium channels recruited during APs. Recently, Marionneau et al. (2012) showed that the expression of Kv4.2, a major constituent of fast inactivating I_A_ channels that is highly expressed in the NAc, decreases in neurons lacking the sodium channel Scn1b subunit in layer 5 cortical pyramidal neurons (Marionneau et al., 2012). Finally, MSNs may adapt to a lack of Scn4b by lowering the expression of the sodium channel pore-forming α subunit.

Voltage-gated calcium channels activated during AP-driven depolarization are likely responsible for dendritic calcium transients as their expression is typically not limited to the soma but also extend to dendrites where they contribute to the active backpropagation of APs along with sodium channels, in a number of brain regions (Markram and Sakmann, 1994; Magee and Johnston, 1995; Kavalali et al., 1997), including the striatum (Carter et al., 2007; Shindou et al., 2011). Nevertheless, they are unlikely to be the only source of calcium modulated in the absence of Scn4b. Indeed, in a previous study, we showed that, upon entering MSNs through L-type calcium channels, an additional pool of calcium is released from intracellular endoplasmic reticulum stores following activation of ryanodine receptors (Ji and Martin, 2012). Therefore, the decrease of the amplitude of dendritic calcium transients in KO and KD mice, that may result from a lesser contribution of VGCC, may be amplified by a weaker calcium-induced calcium release. Perhaps similar amplitudes of calcium signals in primary dendrites in Control and KO mice suggest that if a change of AP threshold is necessary it is not be sufficient, and that other downstream factors may contribute to shaping dendritic calcium signals.

While it remains to be elucidated how a shift of AP threshold impact calcium transients in KO and KD mice, it is likely that the significant drop in calcium in secondary dendrites is responsible for a diminished tLTD. We previously reported that tLTD resulted from the release of cannabinoids binding on CB1 receptors expressed on glutamatergic terminals (Ji and Martin, 2012), confirming previous reports in the striatum (Sadikot et al., 1998; Hoffman et al., 2003; Robbe et al., 2003; Fourgeaud et al., 2004) and other brain regions (Sjostrom et al., 2003; Safo and Regehr, 2005; Peterfi et al., 2012). The role of calcium as a factor critical to tLTD formation is supported by data from the dorsal striatum showing it is inhibited by blocking L-type calcium channels and ryanodine receptors (Plotkin et al., 2013). Furthermore, pairing of uncaged glutamate-mediated EPSPs with APs in dorsal striatum MSNs produced supralinear increase in spine [Ca^2+^]_i_ (Shindou et al., 2011). Interestingly, this effect was only associated with tLTD. Surprisingly, no change of [Ca^2+^]_i_ was observed during tLTP. This study not only highlights the central role played by calcium in mediating APs-mediated tLTD, but also supports the contention that a decrease of calcium transients in dendrites would directly dampen tLTD, as observed here.

Behaviorally, amphetamine-induced locomotion was significantly reduced in KO mice, a phenomenon that is likely the result of a direct involvement of the NAc considering the key role of this brain region in mediating the effects of psychostimulants on locomotion (Kelly et al., 1975; Pijnenburg et al., 1976). Although our study focused on the ventral striatum, we cannot rule out the possibility that the dorsal striatum, a brain region where Scn4b is also highly expressed and that receives dense dopaminergic innervations from the substantia nigra, may also be implicated in the stark behavioral response observed in KO mice. Additionally, in light of the fact that one of amphetamine’s modes of action is to influence dopamine release through the dopamine transporter expressed on presynaptic dopaminergic terminals in striatum (Robertson et al., 2009; Daberkow et al., 2013; Underhill et al., 2014), it is conceivable that the response of KO mice to amphetamine may also indirectly reflect altered excitability of dopaminergic VTA neurons. Indeed, these neurons are innervated by projecting from pyramidal neurons originating in the prefrontal cortex, neurons that also express the Scn4b subunit. Although it is unclear how the Scn4b subunit influences locomotion, inhibition of MSNs firing, as shown in a recent study (Miyazaki et al., 2014), may limit MSNs ability to send information to downstream regions. Alternatively, in light of recent optogenetic-based research in mice showing that fear-related memory can be reactivated and inactivated by LTP and LTD, respectively (Nabavi et al., 2014, #104743), the reduced tLTD probability in NAc MSNs lacking the Scn4b subunit could be interpreted as the inability to erase memories formed during exposure to drugs of abuse, and more specifically when these drugs subvert the neurocircuitry that includes the NAc, to mediate rewards to drugs of abuse (Kelley and Berridge, 2002; Volkow et al., 2008). Of particular interest is the markedly altered expression of mRNA coding for the Scn4b subunit in alcohol-preferring mice and rats (Mulligan et al., 2006; Tabakoff et al., 2008), and more recently in populations of alcoholics (Farris et al., 2014). These studies point to Scn4b as a possible pathway mediating the effects of EtOH on memory related to drugs of abuse rewarding properties. Further studies are needed to better understand how this sodium channel subunit regulates the physiology of MSNs and the role of the NAc in relation with psychostimulants, and more generally with all drugs of abuse.

## Author Contributions

AWL designed and produced the Snc4b shRNA. GEH provided the Scn4b KO mice. XJ performed electrophysiological experiments and analyzed data. SS performed calcium imaging experiments and analyzed data. MG performed behavioral experiments. ART helped design experiments and edited the manuscript. GG provided the adeno associated virus from UMass core viral facility. GEM designed the experiments and wrote the manuscript.

## Conflict of Interest Statement

The authors declare that the research was conducted in the absence of any commercial or financial relationships that could be construed as a potential conflict of interest.

## Supplementary Material

**FIGURE S1 | Details of Scn4b genotype analysis.** Shown are the various loci that were genotyped by Southern blot analysis. Included are relevant restriction sites, probes, and drug selectable markers (Neo, neomycin resistance; TK, thymidine kinase). Magenta and blue triangles represent loxP and frt sites, respectively. The floxed locus w/o Neo can be used to create conditional knockouts. The cre recombined locus lacks Exon 2 and constitutes a global knockout. Not all aspects of diagram are drawn to scale.

**FIGURE S2 | Scn4b ShRNA decreases Scn4b mRNA expression in core NAc MSNs.** **(A)** Image of a slice of tissue stained with GFP antibody indicates location of injection (10×). **(B)** At higher magnification (40×) individual GFP+ cells are clearly visible. In the same slice, double staining with a RFP antibody shows neurons expressing D1 receptors. **(C)** Quantification of mRNA coding for the Scn4b subunit in mice injected with the Control and shRNA-containing virus (shRNA). The symbol ^∗^ indicates *p* < 0.05.

**FIGURE S3 | Effects of Scn4b expression on MSNs resting potential and input resistance.** **(A)** Graph shows resting membrane potential of MSNs from wild type (Wt) and Scn4b knockout mice (KO). Each symbol represents a MSN. Red dotted lines and bars show mean values and SEM, respectively. **(B)** Same as A for MSNs input resistance. **(C)** Current voltage relationship in wild type (wt), knockdown (KD), and knockout (KO) mice. Note the strong effect of Scn4b on the voltage in response to depolarizing current steps.
