# Supplementary figures and images for: The Sodium Channel β4 Auxiliary Subunit Selectively Controls Long-Term Depression in Core Nucleus Accumbens Medium Spiny Neurons
Source: Front Cell Neurosci. 2017 Feb 13;11:17. doi: 10.3389/fncel.2017.00017 (PMC5303751; doi:10.3389/fncel.2017.00017)

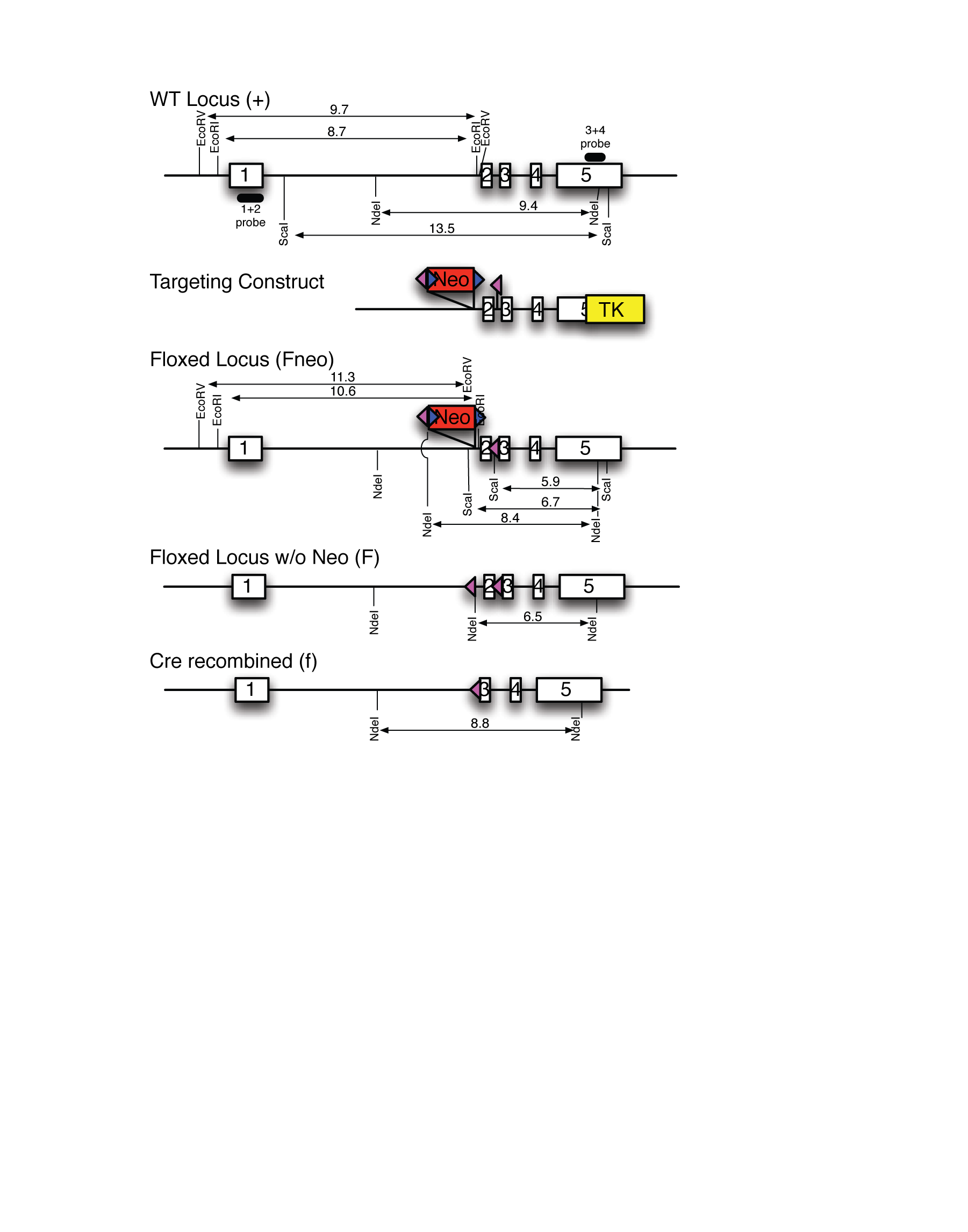

Supplement: Supplementary file 1 [file Image_1.TIF]

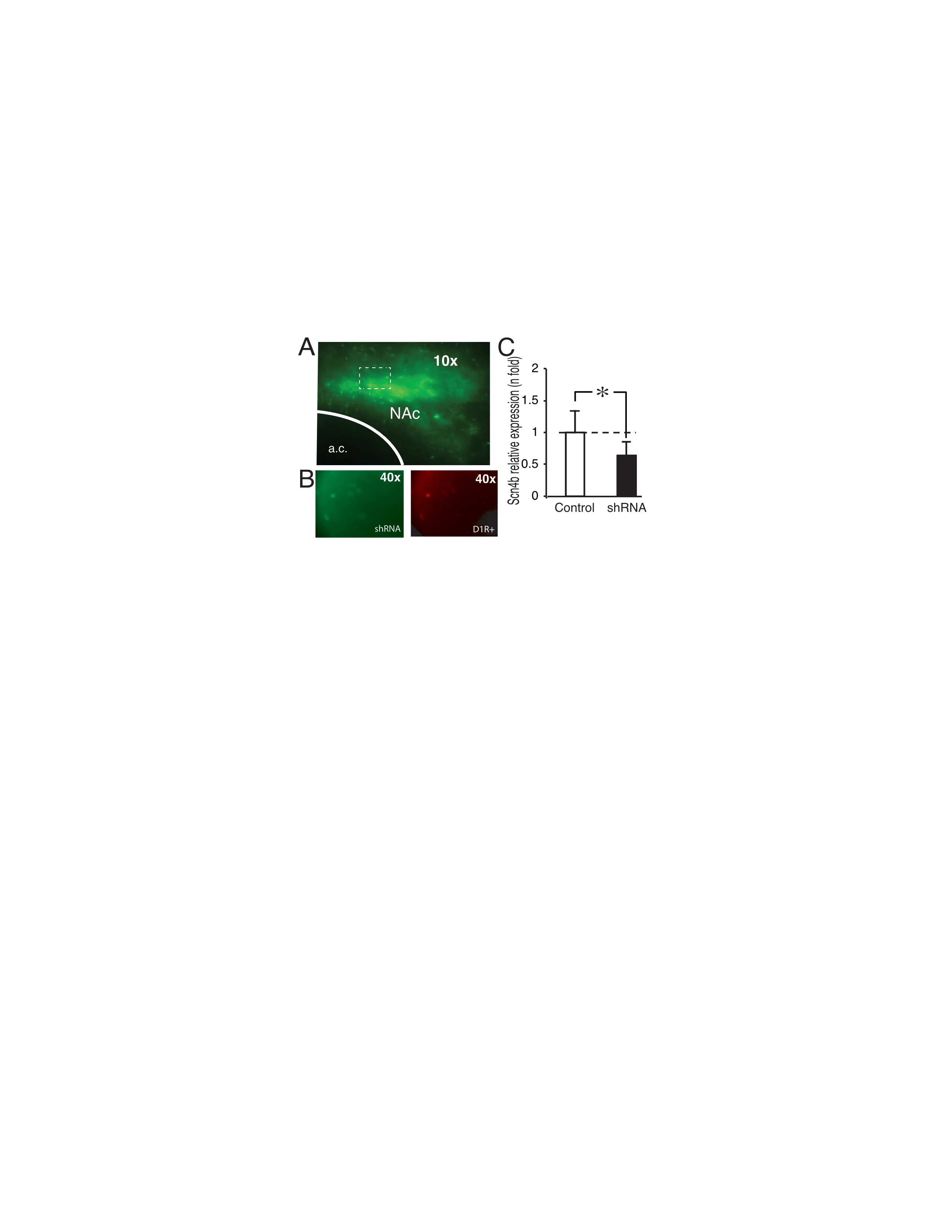

Supplement: Supplementary file 2 [file Image_2.TIFF]

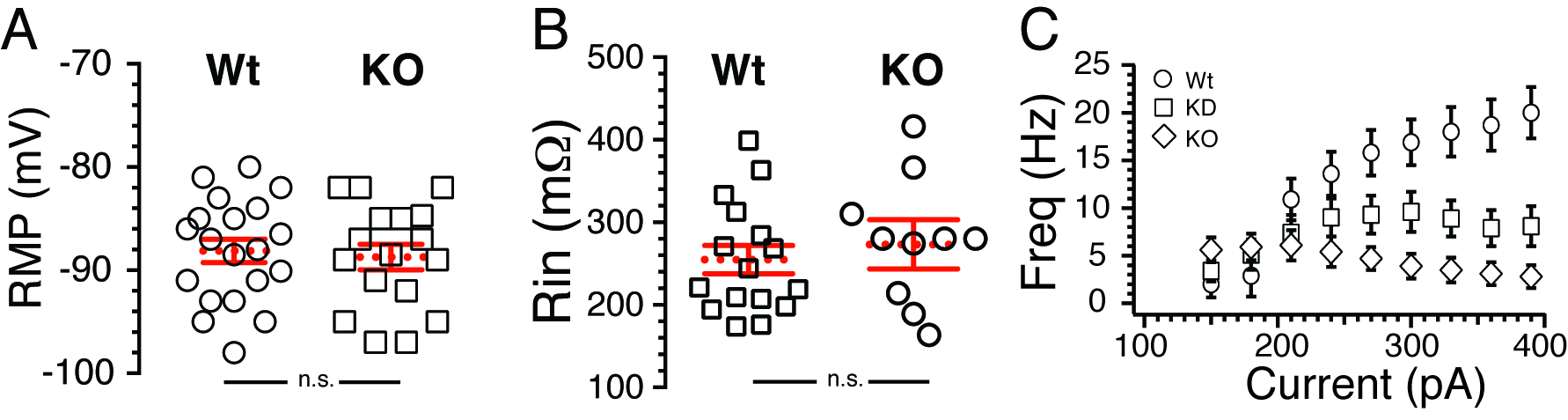

Supplement: Supplementary file 3 [file Image_3.TIF]
